# 

**Published:** 1887-08

**Authors:** 


### OUR ADVERTISERS.

COLDEN'S LIQUID BEEF TONIC.—Dr. John J. Caldwell, of Baltimore, says: "I have used 'Colden's Liquid Beef Tonic' largely in debility, febrile and nervous diseases, and I have found it one of the most reliable of our nutrient tonics now in use or to be found in pharmacy."

DR. SAMUEL E. WOODY, Professor of Chemistry and Public Hygiene and Lecturer on Diseases of Children, Kentucky School of Medicine, at Louisville, on April 8th, said: "papine was used in a case of acute dysentery of unusual severity requiring unusually large doses of opium. The effects of Papine were so purely hypnotic and anodyne that a pound was ordered, and no other form of opium was used during the entire illness. Papine is a pharmaceutical triumph."

RICHMOND AND DANVILLE RAILROAD.—This railroad will carry physicians and their families to Washington and return during the meeting of the International Medical Congress at greatly reduced rates. It offers the advantage over other lines of running solid trains through from Atlanta to Washington without change. This no other line going out of Atlanta can offer. The accommodations on this road are unsurpassed. For rates and sleeping car accommodations write to Mr. C. E. Sargent, passenger agent, Atlanta, Ga.

UTILITY OF CARNRICK'S SOLUBLE FOOD.—In July last there came under my care a lady suffering from parenchymatous rheumatic nephritis. The attack was so acute as to lead to the suspicion of variola, shivering, frequent and terrible lumbar pains, vomiting, but on the third day occurred an œdema, which became general; the urine being examined, removed every doubt as to the diagnosis. In quantity half a litre in twenty-four hours; reaction acid; specific gravity 10.35; reddish-brown color on account of the presence of blood. Tested for albumen it became a solid brown mass almost black. The microscope revealed a great quantity of renal epithelium, numerous blood corpuscles, both red and white, etc. The patient was threatened with pulmonary œdema, an obstinate vomiting of mucus and acid rendered impossible the administration of any remedy to overcome the constipation afflicting the patient from the first, and which prevented any possibility, by the intestinal tract, of compensating for the insufficiency of alimentation. Perspiration was induced, cups were applied to the lumbar region, attempts were made to administer milk, first pure, then deprived of butter by beating, and seasoned with salt or with Carlsbad water, but all were abandoned on account of distressing gastric symptoms being aggravated in the most extraordinary manner. Not knowing where to turn for a dietetic and curative method, it occurred to me that when sick infants were afflicted with gastro-enteritis of the gravest nature, I had always used the Carnrick soluble food with the greatest success. I therefore resolved to try it in this instance. I had a teaspoonful boiled a few moments in a glass of water and gave it hot to my patient, who supported it exceedingly well. I repeated the portion after a few hours. In short, nourished with this food alone and treated with a few subcutaneous injections of hydrochlorate of pilo-carpine, the gastric intolerance diminished, the tongue became clean, the breath less offensive, the quantity of albumen contained in the urine being less, morphologic elements disappeared, phosphites and chlorides re-appeared, and the patient made good recovery.—Dr. Malacrida, in *Gazette Degli Ospitale*, *Milan*, 1886.

